# Interaction analyses of SARS-CoV-2 spike protein based on fragment molecular orbital calculations†

**DOI:** 10.1039/d0ra09555a

**Published:** 2021-01-14

**Authors:** Kazuki Akisawa, Ryo Hatada, Koji Okuwaki, Yuji Mochizuki, Kaori Fukuzawa, Yuto Komeiji, Shigenori Tanaka

**Affiliations:** Department of Chemistry and Research Center for Smart Molecules, Faculty of Science, Rikkyo University 3-34-1 Nishi-ikebukuro Toshima-ku Tokyo 171-8501 Japan fullmoon@rikkyo.ac.jp; Institute of Industrial Science, The University of Tokyo 4-6-1 Komaba Meguro-ku Tokyo 153-8505 Japan; School of Pharmacy and Pharmaceutical Sciences, Hoshi University 2-4-41 Ebara Shinagawa-ku Tokyo 142-8501 Japan; Department of Biomolecular Engineering, Graduate School of Engineering, Tohoku University 6-6-11 Aramaki, Aoba-ku Sendai 980-8579 Japan; Health and Medical Research Institute, AIST Tsukuba Central 6 Tsukuba Ibaraki 305-8566 Japan; Graduate School of System Informatics, Department of Computational Science, Kobe University 1-1 Rokkodai, Nada-ku Kobe 657-8501 Japan

## Abstract

At the stage of SARS-CoV-2 infection in human cells, the spike protein consisting of three chains, A, B, and C, with a total of 3300 residues plays a key role, and thus its structural properties and the binding nature of receptor proteins to host human cells or neutralizing antibodies has attracted considerable interest. Here, we report on interaction analyses of the spike protein in both closed (PDB-ID: 6VXX) and open (6VYB) structures, based on large-scale fragment molecular orbital (FMO) calculations at the level of up to the fourth-order Møller–Plesset perturbation with singles, doubles, and quadruples (MP4(SDQ)). Inter-chain interaction energies were evaluated for both structures, and a mutual comparison indicated considerable losses of stabilization energies in the open structure, especially in the receptor binding domain (RBD) of chain-B. The role of charged residues in inter-chain interactions was illuminated as well. By two separate calculations for the RBD complexes with angiotensin-converting enzyme 2 (ACE2) (6M0J) and B38 Fab antibody (7BZ5), it was found that the binding with ACE2 or antibody partially compensated for this stabilization loss of RBD.

## Abstract 2

Visualized IFIE results seen from chain-B of spike protein.

## Graphical abstract

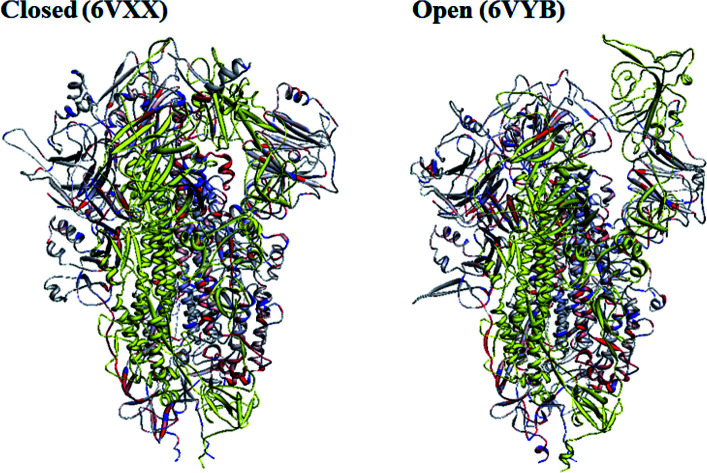

## 1. Introduction

Severe acute respiratory syndrome coronavirus 2 (SARS-CoV-2) is the virus responsible for the 2019 coronavirus disease (COVID-19). First reported in China in December 2019, it has rapidly become a pandemic with devastating effects. The World Health Organization (WHO) Situation Report^1^ records nearly 70 million COVID-19 cases and 1.6 million deaths (December 15, 2020), which numbers are increasing daily. As humans have no direct immunological experience with SARS-CoV-2, we are vulnerable to infection with the virus and the onset of COVID-19. SARS-CoV-2 is highly transmissible; basic reproduction number (*R*_0_) estimates^2,3^ vary between about 2 and 5.

SARS-CoV-2 belongs to the β-coronavirus family and is closely related to SARS-CoV, which was responsible for the 2003 SARS epidemic. As in other coronaviruses, in SARS-CoV-2 the spike protein is a major glycoprotein on the virus surface.^4^ Human SARS coronavirus infections are closely associated with interactions between the viral spike protein and specific human host receptors, such as the angiotensin-converting enzyme 2 (ACE2) receptor.^4,5^ The spike protein is trimeric and has two distinct structural states: prefusion and postfusion. The immune system's recognition of the prefusion state displayed on the virus surface is crucial to mounting an effective immune response. Successful immunization strategies require stable antigens, and elucidation of the interaction between the prefusion state of the viral proteins and the antibodies presents a challenge for vaccine development.

A cryo-EM analysis of the structures of the SARS-CoV-2 spike ectodomain trimer has shown that the spike glycoprotein has two main prefusion conformations: open and closed states.^4^ Subsequent investigations have indicated that the open form is essential for the entry of SARS-CoV-2 into host cells through the binding of its receptor binding domain (RBD)^6^ to the ACE2 receptor. The current model of infection facilitated by the complex of SARS-CoV-2 spike protein and human ACE2 suggests that a reasonable target for structure-based drug discovery might be the disruption of the viral spike protein–ACE2 interface. Thus, a quantitative, structure-based analysis of molecular interactions associated with the SARS-CoV-2 spike protein is necessary to combat COVID-19 by using two main pharmaceutical modalities: vaccination for prevention and drug therapy for patients.

The fragment molecular orbital (FMO) method^7–10^ provides a cost-effective, quantum-chemical tool to evaluate the molecular interactions in biomolecular complexes in an *ab initio* fashion.^11–18^ In ref. 14–17, the influenza virus hemagglutinin (concerning the infections) was investigated in terms of the list of inter-fragment interaction energies (IFIEs)^9–11^ of amino acid residues with antibody or sugar moieties, and useful information such as potential mutation points was successfully derived. Concerning SARS-CoV-2, we have applied the FMO-IFIE analysis to the complexes of 3CL main protease and N3 inhibitor^19^ and of RNA-dependent RNA polymerase with RNA duplex and Remdesivir.^20^ Crucial ligand–residue interactions such as hydrogen bond were revealed for the complex of main protease.^21^ In the present study, we employ a higher-order correlated FMO method^22,23^ to analyze the molecular interactions associated with the spike protein trimers. The interactions among the trimer units (chains A, B, and C) and those between the RBD and the human ACE2 or B38 monoclonal antibody^24^ are analyzed, especially focusing on the difference between the two main structures of spike protein, which are the open and closed forms.

The remainder of this paper is configured as follows. Section 2 summarizes the computational method consisting of the structure preparation of proteins and the scheme of FMO calculations. The first subsection of Section 3 discusses the results of spike protein of the open and closed forms, and the second subsection describes the RBD complexes with ACE2 and B38.

## 2. Computational method

### 2.1. Structure preparation

**Fig. 1:**
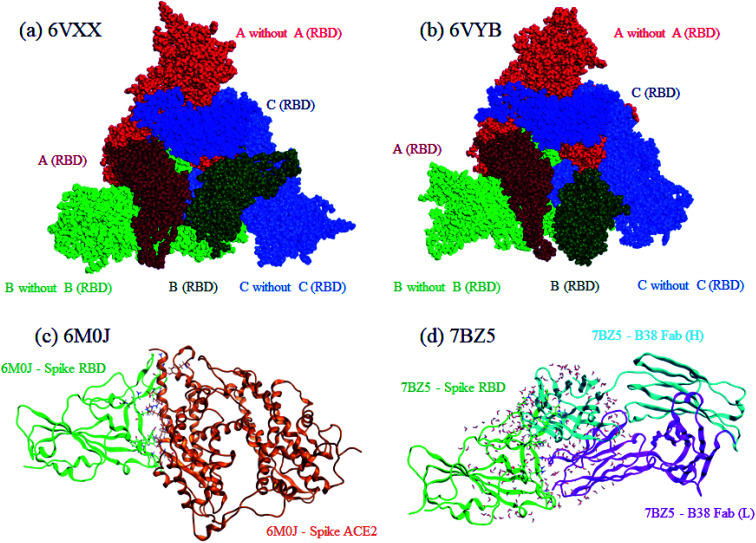
Graphical representations of calculated protein structures. Red, green, and blue indicate chains A, B, and C, respectively, for the closed (a), 6VXX and open (b), 6VYB spike protein. The darker colors identify RBDs in the chains. For the RBD–ACE2 complex (c) 6M0J, RBD and ACE2 are colored green and orange, respectively. Heavy and light chains are colored light-blue and magenta, respectively, for the RBD–B38 Fab complex (d), 7BZ5.

Both closed and open structures of the SARS-CoV-2 spike protein for FMO calculations were prepared from cryo-EM structures, where the corresponding PDB IDs were 6VXX^4^ and 6VYB,^4^ respectively. Note that the latter is assumed to be responsible for infection in human cells. Because the data on both PDB structures lacked several amino acid residues due to their relatively low resolutions, homology modeling was conducted to reproduce the missing parts with the MOE program.^25^ The total number of residues was 3363. The positions of generated hydrogen atoms were optimized also with MOE. For these processed 6VXX (closed) and 6VYB (open) structures, a molecular dynamics (MD)-based relaxation was performed with the AMBER18 program.^26^ These processed structures of 6VXX and 6VYB are illustrated in the upper part of Fig. 1, where chains A, B, and C are colored red, blue, and green, respectively, and the dark-colored parts correspond to RBD (Thr333-Pro527 of each chain). All three RBDs are directed inside in the closed structure (6VXX), whereas the RBD of chain-B is turned outside in the open structure (6VYB). RBDs are crucial in binding with ACE2 located at the cell surface of several antibodies.

For comparative discussion, two complex models of RBD–ACE2 (PDB ID: 6M0J)^5^ and RBD–B38 Fab antibody (7BZ5)^24^ were set up for the FMO calculations. The numbers of processed 6M0J and 7BZ5 residues were 784 and 629, respectively; water molecules in the original PDB records were retained. These RBD complexes are illustrated in the lower part of Fig. 1.

The four above-mentioned protein models (6VXX, 6VYB, 6M0J, and 7BZ5) were subjected to a series of FMO calculations. Further details on the protein structure preparations are described in Section S1 of ESI Part 1.†

### 2.2. FMO calculation

In a previous FMO study of the 3CL main protease with the N3 ligand,^21^ the level of calculations with our ABINIT-MP program^10^ was the second-order Møller–Plesset perturbation (MP2)^27^ at the usual two-body FMO expansion,^28–30^ where a partial renormalization (PR-MP2)^31^ was imposed. The values of IFIE as well as the pair interaction energy decomposition analysis (PIEDA)^32,33^ were used to investigate the chemical situations in pharmacophore. In the present study, beyond-MP2 calculations were employed for more reliable estimations of IFIE/PIEDA.

The third-order MP (MP3) perturbation (in which the interactions among electron pairs are incorporated unlike MP2 (ref. 27 and 34)) was implemented with an integral-direct parallelism in ABINIT-MP;^22^ MP3 has been recently available also in another FMO program PAICS.^35^ Furthermore, ABINIT-MP supports several higher-order treatments^24^ up to the coupled cluster singles and double with perturbative triples (CCSD(T)).^27,34^ In our demonstrative applications,^36,37^ the scaling modification^38^ was utilized to estimate the interaction energies at the CCSD(T) level, where the incremental correlation energies of MP3 and fourth-order MP with singles, doubles, and quadruples (MP4(SDQ))^23^ were halved and added to the MP2 energy, termed MP2.5 (ref. 38) and MP3.5,^37^ respectively. Namely, the scaled estimation is straightforward once MP3 and MP4(SDQ) energies are available.

The FMO-MP3 (ref. 22) calculations (of two-body expansion) were carried out for the spike protein as well as the two RBD complexes on the supercomputer Fugaku managed by the RIKEN Center for Computer Science (R-CCS). In contrast, the FMO-MP4(SDQ)^23^ calculations were done on ITO Subsystem-A at Kyushu University.

Both 6-31G*^39^ and cc-pVDZ^40^ basis sets were employed. Details on the FMO calculations including the conditions of job execution on the two supercomputers are provided in Section S2 of ESI Part 1.†

## 3. Results and discussion

### 3.1. Spike protein

**Fig. 2:**
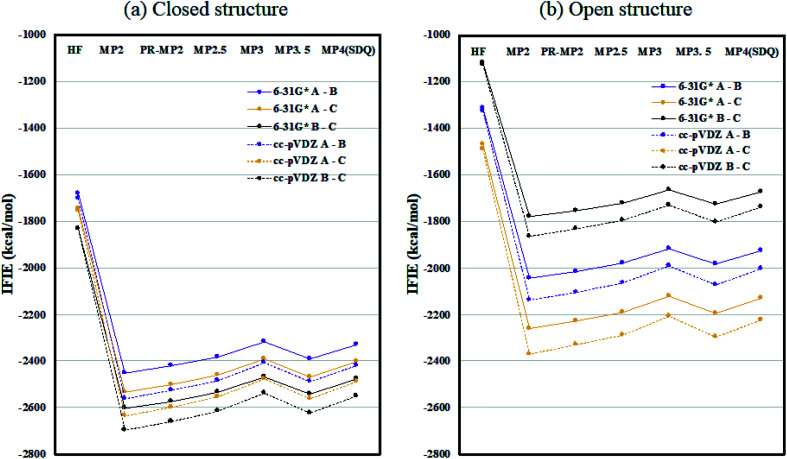
Inter-chain IFIE sums (a) for the closed structure (6VXX) and (b) for the open structure (6VYB). Refer also to the numerical values in Tables S1 and S2.†

Fig. 2 plots the inter-chain IFIE sums (A–B, A–C, and B–C) evaluated at various levels from HF to MP4(SDQ); see the numerical values given in Tables S1 and S2 in ESI Part 2.† First, one can see convergence trends as observed in ref. 36, and the results by MP3.5 should be the most reliable; MP2.5 works well, of course. Comparison between 6-31G* and cc-pVDZ shows that the latter provides larger stabilization energy for a certain chain pair (across levels of calculation), indicating a better ability to describe correlation effects. Hereafter, the values of FMO-MP3.5/cc-pVDZ are primarily used in discussion, as the best estimations accessible currently. The differential energies between the closed (6VXX) and open (6VYB) structures are 418.8 kcal mol^−1^ for the chain A–B pair, 267.3 kcal mol^−1^ for the chain A–C pair, and 821.6 kcal mol^−1^ for the chain B–C pair; refer to Table S3.† The loss of stabilization due to open structure formation for B–C is significantly greater than those for A–C and A–B, and this is consistent with the difference in the contact area of B–C between the closed and open structures, as shown in Fig. 1(a) and (b).

**Fig. 3:**
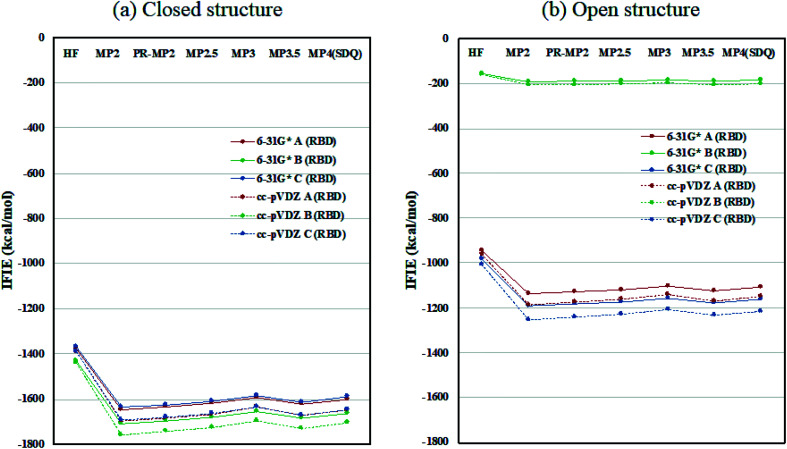
Inter-chain IFIE sums from RBD for (a) the closed structure (6VXX) and (b) the open structure (6VYB). Refer also to Tables S4 and S5.†

Inter-chain IFIE sums from the RBDs are plotted in Fig. 3; refer also to Tables S4 and S5.† In comparison with the closed structure, the RBD of chain-B in the open structure shows a significant loss of stabilization, as much as 1528.2 kcal mol^−1^ (see MP3.5/cc-pVDZ value in Table S6†). The stabilization losses of chain-A RBD and chain-C RBD, which are facing inward, are 504.6 kcal mol^−1^ and 436.0 kcal mol^−1^, respectively, and these values are much smaller than that of chain-B RBD. In summary, the degree of internal stabilizations in the spike protein differs between the closed and open structures, especially in the RBD of chain-B.

**Fig. 4:**
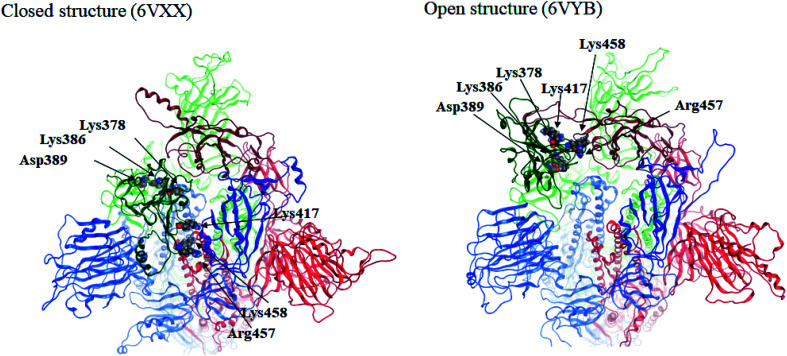
Positions of important residues with differences in IFIEs (more than 150 kcal mol^−1^) between the closed (6VXX) and open (6VYB) structures.

Here we focus on important residues in the RBD of chain-B with large differential IFIEs between the closed (6VXX) and open (6VYB) structures (refer to Table S7†). As expected, those residues whose differential energies are larger than 150 kcal mol^−1^ (Lys386, Lys378, Lys417, Lys458, Asp389, and Arg457) are located in the RBD of chain-B. The positions of these six residues are illustrated in Fig. 4, which clearly shows the large displacements of chain-B RBD from chain-C in the open structure. The roles of charged residues such as Lys are crucial in the inter-chain interactions of the spike protein. The electrostatic interactions can be dominant in total stabilization, and hence the large differences in IFIEs of RBD are consistent. Although the hydration effect might reduce the above energy differences by certain extent (if applicable), fundamental discussion on inter-chain interactions by the present FMO calculations should be valid as in the precedent studies for the hemagglutinin systems of influenza virus.^14–17,22^

### 3.2. RBD complex

**Fig. 5:**
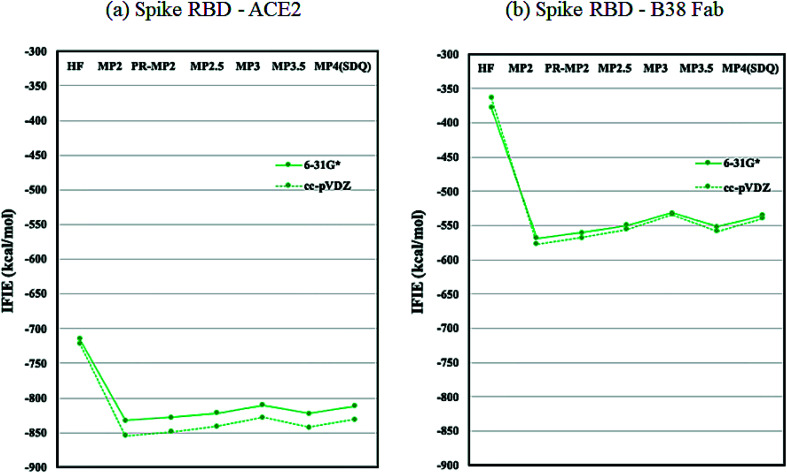
IFIE sums between spike RBD and ACE2 (6M0J) and spike RBD and B38 Fab (7BZ5). Refer also to Tables S8 and S9.†

As discussed in the above subsection, the loss of stabilization in the RBD of chain-B stands out, as the difference with that of chain-A or chain-C is more than 1000 kcal mol^−1^ (Table S6†). We speculated that this loss of RBD may be compensated by the binding with ACE2 or Fab antibody and hence analyzed the protein–protein interaction (PPI) similarly to the FMO-based PPI analyses reported in ref. 41 and 42. Fig. 5 plots the IFIE sums between spike RBD and ACE2 (6M0J) and spike RBD and B38 Fab (7BZ5); the corresponding values are listed in Tables S8 and S9.† The convergence trend across various levels of calculation agrees with the cases of Fig. 2 or 3. The MP3.5/cc-pVDZ value in Table S8† is −842.5 kcal mol^−1^ for ACE2. This roughly corresponds to half the stabilization loss of the chain-B RBD in the open structure (1528.2 kcal mol^−1^), while caution should be paid to the difference in models (including the retention of water molecules) between the full trimer form of 6VXX/6VYB and the RBD–ACE2 complex of 6M0J; refer again to S1 of ESI Part 1.† The largest stabilization is due to Lys417, and all the remaining residues such as Lys457 and Arg458 are of the charged type, as shown in Table S10.† Such a charged residue-driven feature is similar to the situation with the spike protein addressed in the above paragraph (refer again to Table S7†).

The PIEDA results for the important interaction pair of RBD–ACE2 complex (6M0J) are compiled in Table S11† and depicted in Fig. S1.† Lys417 of the RBD provides the largest stabilization with Asp30 in ACE2 (−119.48 kcal mol^−1^), in which the ES contribution dominates. In ref. 5, the importance of the salt-bridge formation of Lys417–Asp30 was suggested, and the present FMO results support this speculation. It is noteworthy that Lys417 has a sizable destabilization with the protonated His34. Other notable residue pairs between RBD and ACE2 are Glu484–Lys31, Tyr449–Asp38, and Phe497–Lys353, in each of which ES contributes dominantly to the stabilization. The importance of Lys417, Tyr449, and Glu484 in the RBD was addressed in ref. 5, 43 and 44 and the present results agree well with them. It is additionally interesting to note that these three residues are unique in SARS-CoV-2; they do not exist in SARS-CoV.^45^ On the ACE2 side, Asp30, Lys31, Asp38, and Lys353 are found to be important in interacting with RBD, in agreement with discussions in other papers.^5,43,44,46^ Lim *et al.* analyzed the 6M0J structure by using a density functional tight binding version of FMO (FMO-DFTB) calculation, with a polarizable continuum model for hydration, and identified several important residues in the interactions between RBD and ACE2.^47^ The present results are in agreement with their hot spot results partially.

Next, the interaction between the RBD and B38 Fab antibody (7BZ5) is discussed; see again Fig. 5 and Table S9.† The IFIE sum is −557.9 kcal mol^−1^ at the MP3.5/cc-pVDZ level, and this value looks to be about one third of the stabilization loss of the chain-B RBD in the open structure. In other words, the binding stabilization of B38 Fab is much smaller than that of ACE2 (−842.5 kcal mol^−1^). The difference may be attributable to the fact that the portions of charged residues in ACE2 and B38 Fab are 22.8% and 15.7%, respectively; the existence of interfacial water molecules in 7BZ5 might also be an origin of difference to a certain extent. As shown in Table S12,† the leading residues of the RBD are of not only the charged type but also the neutral type, and the stabilizations are rather small (refer to Table S10† for ACE2). The PIEDA results (Table S13 and Fig. S2†) indicate that residue pairs of charged–neutral or neutral–neutral types contribute to the total interactions except for the pair of Lys417–Glu98, which gives the most dominant contribution. Thus, Lys417 in RBD can be considered a key player so far. It is notable that the CT and DI contributions are relatively vital in stabilizations. This situation is different from the case of ACE2 complex, in which the ES contribution is dominant due to the charged–charged pairs. Namely, hydrogen bonding and dispersion interaction play essential roles in the stabilization of the RBD–B38 Fab complex. The increments in the IFIE sum from HF to the correlated methods (Fig. 5 and Table S9†) are reasonable because dispersions can be calculated only by inclusion of the electron correlation.

## 4. Summary

In the present study, a series of highly correlated (up to MP4(SDQ) level) FMO calculations were performed for the spike protein and related RBD complexes of SARS-CoV-2. Detailed interaction analyses with the IFIE values revealed sizable differences in inter-chain stabilization energies between the closed and open structures and the important roles of charged residues. In particular, this difference is notable for the RBD of chain-B, which could be responsible for infection in human cells by interacting with ACE2. The results of interaction analyses of the RBD–ACE2 complex suggested that the stabilization loss of the RBD is compensated about halfway by the binding with ACE2. The results also shed light on the roles of charged residues such as Lys417 again. Similar analyses of another RBD complex with B38 Fab antibody indicated that the portion of compensation is smaller than that by ACE2 and that the stabilization from hydrogen bond and dispersion interactions are also important.

Recently, Cao *et al.* designed computationally mini-protein inhibitors for the interaction between RBD and ACE2,^48^ and similar inhibitors were reported by other research groups.^49,50^ Several MD simulations were performed to investigate dynamical behaviors of the spike protein(s) as well.^51–53^ Even though the FMO calculation for the hydrated spike protein is currently impractical to execute, a combination simulation of MD and FMO^54–56^ for the RBD complexes may be one of our future tasks. As denoted in ESI Part 1,† detailed analyses with singular value decomposition (SVD)^57,58^ as a data-science technique have been in progress for a huge amount of PIEDA data on the spike protein, especially to elucidate the importance of inter-chain charged residue pairs in structural differences. Note that another FMO-based work on RBD has recently been reported.^59^ We hope that FMO-based computational approaches will provide useful information for future research into and development of antibodies and inhibitors for the spike protein.^60–66^

## Conflicts of interest

The authors declare no competing financial interest.

## Supplementary Material

RA-011-D0RA09555A-s001

RA-011-D0RA09555A-s002
