# Supplementary material for: Interaction analyses of SARS-CoV-2 spike protein based on fragment molecular orbital calculations
Source: RSC Adv. 2021 Jan 14;11(6):3272–9. doi: 10.1039/d0ra09555a (PMC8694004; doi:10.1039/d0ra09555a)
Supplement: RA-011-D0RA09555A-s001 [file RA-011-D0RA09555A-s001.pdf]

Electronic Supplementary Material (ESI) for RSC Advances.
This journal is © The Royal Society of Chemistry 2021

SUPPLEMENTARY INFORMATION - Part 1

Interaction analyses of SARS-CoV-2 spike protein based on fragment molecular orbital calculations

Kazuki Akisawa[1], Ryo Hatada[1], Koji Okuwaki[1], Yuji Mochizuki[1,2*],

Kaori Fukuzawa[2,3,4], Yuto Komeiji[5], Shigenori Tanaka[6]

1) Department of Chemistry and Research Center for Smart Molecules, Faculty of Science, Rikkyo University, 3-34-1 Nishi-ikebukuro, Toshima-ku, Tokyo 171-8501, Japan

2) Institute of Industrial Science, The University of Tokyo, 4-6-1 Komaba, Meguro-ku, Tokyo 153-8505, Japan

3) School of Pharmacy and Pharmaceutical Sciences, Hoshi University, 2-4-41 Ebara, Shinagawa-ku, Tokyo 142-8501, Japan

4) Department of Biomolecular Engineering, Graduate School of Engineering, Tohoku University, 6-6-11 Aramaki, Aoba-ku, Sendai 980-8579, Japan

5) Health and Medical Research Institute, AIST, Tsukuba Central 6, Tsukuba, Ibaraki 305-8566, Japan

6) Graduate School of System Informatics, Department of Computational Science, Kobe University, 1-1 Rokkodai, Nada-ku, Kobe 657-8501, Japan

For RSC Adv. - 2021/1/12, JST

Email: fullmoon@rikkyo.ac.jp (Y. Mochizuki: CA[*])

S1. Structure preparations of proteins

The PDB IDs of the closed and open structures of the spike protein were 6VXX[1] (resolution 2.80 Å) and 6VYB[1] (3.20 Å), respectively. These PDB structure data lack a significant number of amino acid residues due to relatively low resolutions of cryo-EM structures; no internal water molecule was found within the three protein chains. Hence, the MOE[2] utility was used to compensate the missing parts with homology modeling and also to add hydrogen atoms. With the AMBER10:EHT force field,[3] the positions of hydrogen atoms were first optimized and then other parts were relaxed under the harmonic restraint on the main chains. Charged models were assigned to the N- and C-termini of the protein chains. The total number of residues was 3363 for both processed structures, and the numbers of atoms were 51675 for 6VXX and 51671 and 6VYB; these small differences due to the homology modeling could not have affected the main results.

For the processed structures of 6VXX and 6VYB, a classical molecular dynamics (MD)-based relaxation with the ff14SB force field[4] was performed by using the AMBER18 program.[5] We performed the structure relaxation while restraining the main chain atoms to their original positions with a harmonic potential: Step (1) thermal elevation from 0 K to 50 K (50 ps), Step (2) thermal equilibration (1 ns), Step (3) thermal annealing at 10 K (100 ps), and Step (4) re-optimization on MD structure of step (3).

These MD calculations were performed in vacuum without a periodic boundary condition on the TSUBAME3.0 supercomputer. The time step was set to 1 fs without bond constraints.

The resolutions of 6M0J (RBD[6] with ACE2)[7] and 7BZ5 (RBD with B38 Fab antibody)[8] were 2.45 Å and 1.84 Å, respectively, and these values were better than those of 6VXX and 6VYB. Standard processing with MOE was used: the attachment of hydrogen atoms and the optimization with the AMBER10:EHT force field. The N- and C-termini of the RBD were set to neutral because the RBD is part of a protein chain. The numbers of total residues of 6M0J and 7BZ5 were 784 and 629, respectively. The number of water molecules retained from the original PDB records were 80 (fully kept) for 6M0J and 305 (reduced from 519) for 7BZ5. In 6M0J, no water molecule was found in the interfacial region between RBD and ACE2, and all the water molecules were distributed over protein surfaces. In 7BZ5, however, there existed a number of water molecules at both interfacial and surrounding regions; hence, we removed those farther than 30 Å from Lys417 as a key residue of salt-bridge.

Ideally, the inclusion of hydration effect with water molecules as well as counter ions is desirable for the structure modeling of spike protein and RDB complexes, based on MD simulations. However, the execution of fragment molecular orbital (FMO)

calculation for these systems was far from practical, because a total number of fragments in such an explicitly hydrated structure would reach tens of thousands. In Refs. 9-12, the influenza virus hemagglutinin was investigated by the FMO interaction analyses, where no hydration effect was taken into account as in the case of present study. In spite of this restriction in the structure modeling of hemagglutinin complexes, the nature of interactions between protein chains and Fab antibody or sugar moieties as well as the potential mutation points were successfully revealed through discussions of relative (not absolute) IFIE values. We would thus consider that the present prepared protein structures are acceptable, as long as caution should be paid in discussion for the existence of water molecules for separated RBD complexes (6M0J and 7BZ5) in comparison with full trimer chain models (6VXX and 6VYB).

References for S1

1. A. C. Walls, Y.-J. Park, M. A. Tortorici, A. Wall, A. T. McGuire and D. Veesler, *Cell*, 2020, **181**, 281-292.e6.

2. Molecular Operating Environment (MOE) (2019.01), Chemical Computing Group ULC, 1010 Sherbooke St. West, Suite #910, Montreal, QC, Canada, H3A 2R7, 2021.
3. D. A. Case, T. E. Cheatham III, T. Darden, H. Gohlke, R. Luo, K. M. Merz Jr., A. Onufriev, C. Simmerling, B. Wang and R. J. Woods, *J. Comput. Chem.*, 2005, **26**, 1668–1688.
4. J. A. Maier, C. Martinez, K. Kasavajhala, L. Wickstrom, K. E. Hauser and C. Simmerling, *J. Chem. Theory Comput.*, 2015, **11**, 3696–3713.
5. D.A. Case, I.Y. Ben-Shalom, S.R. Brozell, D.S. Cerutti, T.E. Cheatham, III, V.W.D. Cruzeiro, T.A. Darden, R.E. Duke, D. Ghoreishi, M.K. Gilson, H. Gohlke, A.W. Goetz, D. Greene, R Harris, N. Homeyer, Y. Huang, S. Izadi, A. Kovalenko, T. Kurtzman, T.S. Lee, S. LeGrand, P. Li, C. Lin, J. Liu, T. Luchko, R. Luo, D.J. Mermelstein, K.M. Merz, Y. Miao, G. Monard, C. Nguyen, H. Nguyen, I. Omelyan, A. Onufriev, F. Pan, R. Qi, D.R. Roe, A. Roitberg, C. Sagui, S. Schott-Verdugo, J. Shen, C.L. Simmerling, J. Smith, R. SalomonFerrer, J. Swails, R.C. Walker, J. Wang, H. Wei, R.M. Wolf, X. Wu, L. Xiao, D.M. York and P.A. Kollman, AMBER 2018, University of California, San Francisco, 2018.
6. F. Li, W. Li, M. Farzan and S. C. Harrison, *Science.*, 2005, **309**, 1864–1868.

7. J. Lan, J. Ge, J. Yu, S. Shan, H. Zhou, S. Fan, Q. Zhang, X. Shi, Q. Wang, L. Zhang and X. Wang, *Nature*, 2020, **581**, 215–220.
8. Y. Wu, F. Wang, C. Shen, W. Peng, D. Li, C. Zhao, Z. Li, S. Li, Y. Bi, Y. Yang, Y. Gong, H. Xiao, Z. Fan, S. Tan, G. Wu, W. Tan, X. Lu, C. Fan, Q. Wang, Y. Liu, C. Zhang, J. Qi, G. F. Gao, F. Gao and L. Liu, *Science.*, 2020, **368**, 1274–1278.
9. T. Iwata, K. Fukuzawa, K. Nakajima, S. Aida-Hyugaji, Y. Mochizuki, H. Watanabe and S. Tanaka, *Comput. Biol. Chem.*, 2008, **32**, 198–211.
10. S. Anzaki, C. Watanabe, K. Fukuzawa, Y. Mochizuki and S. Tanaka, *J. Mol. Graph. Model.*, 2014, **53**, 48–58.
11. K. Takematsu, K. Fukuzawa, K. Omagari, S. Nakajima, K. Nakajima, Y. Mochizuki, T. Nakano, H. Watanabe and S. Tanaka, *J. Phys. Chem. B*, 2009, **113**, 4991–4994.
12. A. Yoshioka, K. Fukuzawa, Y. Mochizuki, K. Yamashita, T. Nakano, Y. Okiyama, E. Nobusawa, K. Nakajima and S. Tanaka, *J. Mol. Graph. Model.*, 2011, **30**, 110–119.

S2. Fragment molecular orbital calculations

In the present study, the ABINIT-MP program[1] was adopted for all of the fragment molecular orbital (FMO) calculations at the conventional two-body expansion.[1-4] The proteins were fragmented in the usual residue-by-residue manner. A series of interaction analyses were performed based upon the lists of inter-fragment interaction energy (IFIE),[1,5] which is also called pair interaction energy (PIE)[4] in GAMESS-US.[6] The decomposition analysis of PIE (PIEDA)[7,8] was used to understand the nature of interactions in more detail when necessary.[9-15] In PIEDA, the electrostatic (ES), Pauli exchange (EX), and charge transfer (CT) energies are evaluated at the Hartree-Fock (HF) level.[16] The remaining contribution from dispersion interaction (DI) is usually calculated at the second-order Møller-Plesset perturbation (MP2) level[16,17] as a standard beyond-HF recipe. The π/π and CH/π[18] interactions are of the dispersion type, and HF hardly describes these stabilizations.

It is known that MP2 overestimates the interaction energy, but that this overestimation can be remedied by higher-order correlated treatments in which electron pair - pair interactions are incorporated.[16] Manifestly, the coupled cluster singles and doubles with perturbative triples (CCSD(T))[17] is the best recipe; however, its cost of computation for larger systems is too high. Note that the formal cost orders of MP2, the third-order MP

(MP3), and CCSD(T) are non-iterative N^5, non-iterative N^6, and iterative N^6 plus non-iterative N^7 (N is the number of MOs in a given system), respectively.[17] The naive MP3 evaluation rather underestimates interaction energies, especially dispersion-type interactions. Pitonak et al.[19] thus proposed a scaling scheme named MP2.5 in which the MP3 incremental correlation energy to be added to the MP2 energy is halved by considering the convergence behavior toward the final value by CCSD(T). In fact, MP2.5 scaling favorably improved the evaluation of interaction energies.[20,21] In Ref. 21, besides MP2.5, we attempted its extended version, namely, MP3.5, with the halved incremental correlation energy by the MP4[17] with singles, doubles, and quadruples (MP4(SDQ)). Our MP3.5 scheme was different from Katron's proposal,[22] in which the full MP4 included triples with N^7 cost. MP3.5 showed slightly better convergence performance than MP2.5 did.

The 6-31G*[23] and cc-pVDZ[24] basis sets were used in the present FMO calculations. The basis set dependence on IFIE values was previously tested for some archetype cases of dispersion-driven interactions at the MP2 level.[25] Ref. 25 showed a better flexibility of cc-pVDZ over 6-31G* and also a necessity of cc-pVTZ[24] or cc-pVQZ basis sets for accurate estimations. Unfortunately, the FMO calculation with cc-pVTZ for the spike protein is prohibitive even with currently available supercomputers of the highest level

performance (addressed below). We would thus have a stance that the cc-pVDZ basis set in conjunction with the MP3.5 scaling estimation is a practical choice of best-effort at the present time.

In ABINIT-MP, the MP3 calculation has been supported with a dedicated module,[26] and several higher-order methods up to CCSD(T) have been available with a general beyond-MP2 module[27] as well; these modules require more memory spaces than the MP2 module does. The FMO-MP3 (with PIEDA option[8]) jobs[26] were executed on the Fugaku supercomputer operated by the RIKEN Center for Computational Science (R-CCS). Under an OpenMP/MPI hybrid parallelization, a total of 48 cores (A64FX) with a 16 GB shared memory setting (thread parallelization) per node were utilized for the internal processing of the fragment monomer or dimer. A total of 3072 nodes (for process parallelization) were employed; a total number of cores was thus as many as 147456. In contrast, the FMO-MP4(SDQ) jobs[27] were run on ITO Subsystem-A at Kyushu University under a dedicated usage of computing resources. A memory space of 75 GB and 18 cores (Intel Xeon Gold 6154) were assigned to the internal fragment processing with an OpenMP thread parallelism, and a total of 2000 MPI processes were invoked for the list of fragment monomers and dimers; a total number of used cores was 36000.

On Fugaku, the FMO-MP2 jobs[28-30] with a partial renormalization (PR-MP2)[31] option

were imposed as well. PR-MP2 reduced the trend of the overestimation of IFIE values, within the same cost of MP2. The correlation energies of IFIE or DI energies in PIEDA were then evaluated by the correlation energies of MP2, PR-MP2, MP2.5, MP3, MP3.5, and MP4(SDQ).

Dimers consisting of distant monomers (> 2 sum of van der Waals contact radii) are usually treated by the dimer electrostatic approximation (Dimer-ES)[32], by which both HF and correlated calculations are skipped. Dimer-ES works well for medium-sized proteins (a few hundred residues). However, the cost of integral evaluations grows rapidly when the number of residues becomes a few thousand. In the ABINIT-MP program, the continuous multipole moment (CMM) evaluation[33] has been available to accelerate the Dimer-ES step (typically 20 times). The difference in the total HF energy can be maintained as small as 1.0×10^{-4} au, as long as the proper threshold is set to define the truly far region (> 5 sum of van der Waals contact radii). The Dimer-ES CMM option was thus used in the present calculations for the spike protein and the RBD complexes.

The numbers of fragments of 6VXX (closed) and 6VYB (open) were 3327 and 3328, respectively. The bridged Cys-Cys was merged into a single fragment. The timings of FMO-MP3[26] jobs for 6VXX were 1.9 h for the 6-31G* basis and 3.4 h for the cc-pVDZ basis on Fugaku. Interestingly, the FMO-MP2 jobs of 6-31G* and cc-pVDZ were

completed in 0.9 h and 1.7 h, respectively. Namely, the incremental factor from MP2 to MP3 was as small as only 2, demonstrating the utility and practicality of FMO-MP3 calculations on Fugaku; the formal scaling costs of N^5 versus N^6 are not necessarily valid for FMO calculations with massive computing facilities.[26] On the other hand, the timings of FMO-MP4(SDQ)[27] jobs for 6VXX were 5.4 h (6-31G*) and 8.6 h (cc-pVDZ), respectively, on ITO Subsystem-A. The jobs for 6VYB were processed in timings similar to those for 6VXX.

6M0J (RBD - ACE2) and 7BZ5 (RBD - B38 Fab) had 864 and 926 fragments, respectively, because a number of water molecules (in the interfacial region) were included besides proteins. The timings of FMO-MP3/cc-pVDZ jobs for 6M0J and 7BZ5 on 768 nodes of Fugaku (48 cores per fragment) were 2.5 h and 2.1 h, respectively. With 1000 processes (18 cores per fragment for threads) of ITO Subsystem-A, the FMO-MP4(SDQ)/cc-pVDZ jobs for 6M0J and 7BZ5 were finished in 4.3 h and 3.2 h, respectively.

The PIEDA results were excessively large for the spike protein (6VXX and 6VYB), and concise discussions are rather hard. We thus decided to show the corresponding data in another paper, in which the technique of singular value decomposition (SVD)[34,35] is employed to extract essential features. In the present paper, a couple of RBD complexes

(6M0J and 7BZ5) are discussed with the help of PIEDA; refer to Tables S11 and S13 as well as Figures S1 and S2 in Supplemental Information Part 2.

The system and compiler improvements to Fugaku have been ongoing, although its benchmark performance proved to be the world's fastest as of November 2020 as well as June 2020.[36] The above-noted timing results of FMO-MP3 were obtained in evaluation environments in the trial phase of Fugaku[37] and thus were tentative; the performance, power, and other attributes at the start of its public use operation from fiscal year 2021 (which in Japan begins April 1, 2021) cannot be guaranteed. Additionally, a direct comparison of job timing data between Fugaku and ITO Subsystem-A was unavailable because the computational environments of these two supercomputers and the modules used (for MP3 and MP4(SDQ)) differed from each other.

As described so far, the present FMO calculations with ABINIT-MP[1] were performed at up to the MP4(SDQ) level, based on the usage of two supercomputers, Fugaku and ITO Subsystem-A. With GAMESS-US,[6] Katouda et al. carried out a resolution-of-identity MP2 (RI-MP2) type FMO computation for an influenza protein (968 residues), by using 86016 cores of the K-computer (one before Fugaku), and its job time was only 0.3 h at that time.[38] Massively parallel computing resources with over ten thousand cores on supercomputers should provide a power to make large-scale FMO calculations as in the

cases of regular MO calculations.[39,40]

References for S2

1. S. Tanaka, Y. Mochizuki, Y. Komeiji, Y. Okiyama and K. Fukuzawa, *Phys. Chem. Chem. Phys.*, 2014, **16**, 10310–10344.
2. D. G. Fedorov and K. Kitaura, The Fragment Molecular Orbital Method: Practical Applications to Large Molecular Systems, CRC Press, London, 2009.
3. K. Kitaura, E. Ikeo, T. Asada, T. Nakano and M. Uebayasi, *Chem. Phys. Lett.*, 1999, **313**, 701–706.
4. D. G. Fedorov, T. Nagata and K. Kitaura, *Phys. Chem. Chem. Phys.*, 2012, **14**, 7562–7577.
5. S. Amari, M. Aizawa, J. Zhang, K. Fukuzawa, Y. Mochizuki, Y. Iwasawa, K. Nakata, H. Chuman and T. Nakano, *J. Chem. Inf. Model.*, 2006, **46**, 221–230.
6. D. G. Fedorov, *WIREs Comput. Mol. Sci.*, 2017, **7**, e1322.
7. D. G. Fedorov and K. Kitaura, *J. Comput. Chem.*, 2007, **28**, 222–237.
8. T. Tsukamoto, K. Kato, A. Kato, T. Nakano, Y. Mochizuki and K. Fukuzawa, *J. Comput. Chem. Japan*, 2015, **14**, 1–9.

9. C. Watanabe, H. Watanabe, K. Fukuzawa, L. J. Parker, Y. Okiyama, H. Yuki, S. Yokoyama, H. Nakano, S. Tanaka and T. Honma, *J. Chem. Inf. Model.*, 2017, **57**, 2996–3010.
10. Y. Komeiji, Y. Okiyama, Y. Mochizuki and K. Fukuzawa, *Bull. Chem. Soc. Jpn.*, 2018, **91**, 1596–1605.
11. T. Tokiwa, S. Nakano, Y. Yamamoto, T. Ishikawa, S. Ito, V. Sladek, K. Fukuzawa, Y. Mochizuki, H. Tokiwa, F. Misaizu and Y. Shigeta, *J. Chem. Inf. Model.*, 2019, **59**, 25–30.
12. Y. Okiyama, C. Watanabe, K. Fukuzawa, Y. Mochizuki, T. Nakano and S. Tanaka, *J. Phys. Chem. B*, 2019, **123**, 957–973.
13. A. Heifetz, I. Morao, M. M. Babu, T. James, M. W. Y. Southey, D. G. Fedorov, M. Aldeghi, M. J. Bodkin and A. Townsend-Nicholson, *J. Chem. Theory Comput.*, 2020, **16**, 2814–2824.
14. R. Hatada, K. Okuwaki, Y. Mochizuki, Y. Handa, K. Fukuzawa, Y. Komeiji, Y. Okiyama and S. Tanaka, *J. Chem. Inf. Model.*, 2020, **60**, 3593–3602.
15. K. Kato, T. Honma and K. Fukuzawa, *J. Mol. Graph. Model.*, 2020, **100**, 107695.
16. A. Szabo, N. S. Ostlund, Modern Quantum Chemistry, MacMillan, New York, 1982.

17. I. Shavitt and R. J. Bartlett, Many-body Methods in Chemistry and Physics, Cambridge Univeristy Press, Cambridge, 2009.

18. Y. Umezawa, S. Tsuboyama, K. Honda, J. Uzawa and M. Nishio, *Bull. Chem. Soc. Jpn.*, 1998, **71**, 1207–1213.

19. M. Pitoňák, P. Neogrády, J. Černý, S. Grimme and P. Hobza, *ChemPhysChem*, 2009, **10**, 282–289.

20. Y. Okiyama, K. Fukuzawa, H. Yamada, Y. Mochizuki, T. Nakano and S. Tanaka, *Chem. Phys. Lett.*, 2011, **509**, 67–71.

21. H. Yamada, Y. Mochizuki, K. Fukuzawa, Y. Okiyama and Y. Komeiji, *Comput. Theor. Chem.*, 2017, **1101**, 46–54.

22. A. Karton and L. Goerigk, *J. Comput. Chem.*, 2015, **36**, 622–632.

23. J. B. Foresman and A. Frisch, Exploring chemistry with electronic structure methods, 2nd edn, Gaussian Inc., Pittsburgh, 1996.

24. T. H. Dunning, *J. Chem. Phys.*, 1989, **90**, 1007–1023.

25. T. Ishikawa, Y. Mochizuki, S. Amari, T. Nakano, S. Tanaka and K. Tanaka, *Chem. Phys. Lett.*, 2008, **463**, 189–194.

26. Y. Mochizuki, K. Yamashita, K. Fukuzawa, K. Takematsu, H. Watanabe, N. Taguchi, Y. Okiyama, M. Tsuboi, T. Nakano and S. Tanaka, *Chem. Phys. Lett.*, 2010, **493**, 346–352.

27. Y. Mochizuki, K. Yamashita, T. Nakano, Y. Okiyama, K. Fukuzawa, N. Taguchi and S. Tanaka, *Theor. Chem. Acc.*, 2011, **130**, 515–530.

28. Y. Mochizuki, S. Koikegami, T. Nakano, S. Amari and K. Kitaura, *Chem. Phys. Lett.*, 2004, **396**, 473–479.

29. Y. Mochizuki, T. Nakano, S. Koikegami, S. Tanimori, Y. Abe, U. Nagashima and K. Kitaura, *Theor. Chem. Acc.*, 2004, **112**, 442–452.

30. Y. Mochizuki, K. Yamashita, T. Murase, T. Nakano, K. Fukuzawa, K. Takematsu, H. Watanabe and S. Tanaka, *Chem. Phys. Lett.*, 2008, **457**, 396–403.

31. C. E. Dykstra and E. R. Davidson, *Int. J. Quantum Chem.*, 2000, **78**, 226–236.

32. T. Nakano, T. Kaminuma, T. Sato, K. Fukuzawa, Y. Akiyama, M. Uebayasi and K. Kitaura, *Chem. Phys. Lett.*, 2002, **351**, 475–480.

33. T. Nakano, K. Yamashita, K. Segawa, Y. Okiyama, C. Watanabe, K. Fukuzawa, S. Tanaka and Y. Mochidzuki, *J. Comput. Aided Chem.*, 2012, **13**, 44–50.

34. K. Maruyama, Y. Sheng, H. Watanabe, K. Fukuzawa and S. Tanaka, *Comput. Theor. Chem.*, 2018, **1132**, 23–34.

35. S. Tanaka, C. Watanabe, T. Honma, K. Fukuzawa, K. Ohishi and T. Maruyama, *J. Mol. Graph. Model.*, 2020, **100**, 107650.

36. https://www.top500.org/ (access confirmed on 2021/1/7).

37. https://www.r-ccs.riken.jp/en/topics/fugaku-coronavirus.html (access confirmed on 2021/1/7).

38. M. Katouda, T. Nakajima and S. Nagase, *Proceedings of JSST 2012.*, **2012**, 338–343.

39. E. Aprà, E. J. Bylaska, W. A. de Jong, N. Govind, K. Kowalski, T. P. Straatsma, M. Valiev, H. J. J. van Dam, Y. Alexeev, J. Anchell, V. Anisimov, F. W. Aquino, R. Atta-Fynn, J. Autschbach, N. P. Bauman, J. C. Becca, D. E. Bernholdt, K. Bhaskaran-Nair, S. Bogatko, P. Borowski, J. Boschen, J. Brabec, A. Bruner, E. Cauët, Y. Chen, G. N. Chuev, C. J. Cramer, J. Daily, M. J. O. Deegan, T. H. Dunning, M. Dupuis, K. G. Dyall, G. I. Fann, S. A. Fischer, A. Fonari, H. Früchtl, L. Gagliardi, J. Garza, N. Gawande, S. Ghosh, K. Glaesemann, A. W. Götz, J. Hammond, V. Helms, E. D. Hermes, K. Hirao, S. Hirata, M. Jacquelin, L. Jensen, B. G. Johnson, H. Jónsson, R. A. Kendall, M. Klemm, R. Kobayashi, V. Konkov, S. Krishnamoorthy, M. Krishnan, Z. Lin, R. D. Lins, R. J. Littlefield, A. J. Logsdail, K. Lopata, W. Ma, A. V Marenich, J. Martin del Campo, D. Mejia-

Rodriguez, J. E. Moore, J. M. Mullin, T. Nakajima, D. R. Nascimento, J. A. Nichols, P. J. Nichols, J. Nieplocha, A. Otero-de-la-Roza, B. Palmer, A. Panyala, T. Pirojsirikul, B. Peng, R. Peverati, J. Pittner, L. Pollack, R. M. Richard, P. Sadayappan, G. C. Schatz, W. A. Shelton, D. W. Silverstein, D. M. A. Smith, T. A. Soares, D. Song, M. Swart, H. L. Taylor, G. S. Thomas, V. Tipparaju, D. G. Truhlar, K. Tsemekhman, T. Van Voorhis, Á. Vázquez-Mayagoitia, P. Verma, O. Villa, A. Vishnu, K. D. Vogiatzis, D. Wang, J. H. Weare, M. J. Williamson, T. L. Windus, K. Woliński, A. T. Wong, Q. Wu, C. Yang, Q. Yu, M. Zacharias, Z. Zhang, Y. Zhao and R. J. Harrison, *J. Chem. Phys.*, 2020, **152**, 184102.

40. C. Peng, C. A. Lewis, X. Wang, M. C. Clement, K. Pierce, V. Rishi, F. Pavošević, S. Slattery, J. Zhang, N. Teke, A. Kumar, C. Masteran, A. Asadchev, J. A. Calvin and E. F. Valeev, *J. Chem. Phys.*, 2020, **153**, 44120.
