# Supplementary material for: Interaction analyses of SARS-CoV-2 spike protein based on fragment molecular orbital calculations
Source: RSC Adv. 2021 Jan 14;11(6):3272–9. doi: 10.1039/d0ra09555a (PMC8694004; doi:10.1039/d0ra09555a)
Supplement: RA-011-D0RA09555A-s002 [file RA-011-D0RA09555A-s002.pdf]

Electronic Supplementary Material (ESI) for RSC Advances.
This journal is © The Royal Society of Chemistry 2021

SUPPLEMENTARY INFORMATION - Part 2

Interaction analyses of SARS-CoV-2 spike protein based on fragment molecular orbital calculations

Kazuki Akisawa[1], Ryo Hatada[1], Koji Okuwaki[1], Yuji Mochizuki[1,2*],

Kaori Fukuzawa[2,3,4], Yuto Komeiji[5], Shigenori Tanaka[6]

1) Department of Chemistry and Research Center for Smart Molecules, Faculty of Science, Rikkyo University, 3-34-1 Nishi-ikebukuro, Toshima-ku, Tokyo 171-8501, Japan

2) Institute of Industrial Science, The University of Tokyo, 4-6-1 Komaba, Meguro-ku, Tokyo 153-8505, Japan

3) School of Pharmacy and Pharmaceutical Sciences, Hoshi University, 2-4-41 Ebara, Shinagawa-ku, Tokyo 142-8501, Japan

4) Department of Biomolecular Engineering, Graduate School of Engineering, Tohoku University, 6-6-11 Aoba, Aramaki, Aoba-ku, Sendai 980-8579, Japan

5) Health and Medical Research Institute, AIST, Tsukuba Central 6, Tsukuba, Ibaraki 305-8566, Japan

6) Graduate School of System Informatics, Department of Computational Science, Kobe University, 1-1 Rokkodai, Nada-ku, Kobe 657-8501, Japan

For RSC Adv. - 2021/1/7, JST

Email: fullmoon@rikkyo.ac.jp (Y. Mochizuki: CA[*])

Table S1. Inter-chain IFIE sums for the closed structure (6VXX). Units in kcal/mol.

Method	6-31G*			cc-pVDZ		
	A - B	A - C	B - C	A - B	A - C	B - C
HF	-1683.1	-1745.1	-1832.5	-1700.8	-1755.3	-1832.4
MP2	-2451.2	-2531.3	-2603.3	-2560.2	-2635.6	-2695.0
PR-MP2	-2421.0	-2500.4	-2572.7	-2523.1	-2597.8	-2658.1
MP2.5	-2384.4	-2461.5	-2535.0	-2482.5	-2555.2	-2616.2
MP3	-2317.6	-2391.7	-2466.7	-2404.9	-2474.9	-2537.3
MP3.5	-2389.6	-2466.6	-2540.1	-2489.6	-2562.1	-2623.2
MP4(SDQ)	-2328.1	-2402.0	-2477.0	-2419.0	-2488.7	-2551.4

Table S2. Inter-chain IFIE sums for the open structure (6VYB). Units in kcal/mol.

Method	6-31G*			cc-pVDZ		
	A - B	A - C	B - C	A - B	A - C	B - C
HF	-1314.8	-1468.6	-1116.5	-1328.0	-1487.6	-1125.0
MP2	-2042.7	-2260.5	-1779.7	-2138.2	-2368.4	-1863.3
PR-MP2	-2013.6	-2229.1	-1753.1	-2103.0	-2330.6	-1831.5
MP2.5	-1979.2	-2190.2	-1722.0	-2064.8	-2287.8	-1797.0
MP3	-1915.6	-2119.9	-1664.4	-1991.4	-2207.3	-1730.7
MP3.5	-1984.0	-2195.6	-1725.7	-2070.8	-2294.9	-1801.6
MP4(SDQ)	-1925.2	-2130.8	-1671.8	-2003.4	-2221.3	-1739.9

Table S3. Differential inter-chain IFIE sums between the closed structure (6VXX) and open structure (6VYB). Positive values indicate destabilization for the latter. Units in kcal/mol.

Method	6-31G*			cc-pVDZ		
	A - B	A - C	B - C	A - B	A - C	B - C
HF	368.3	276.5	716.0	372.8	267.7	707.4
MP2	408.4	270.8	823.6	422.0	267.2	831.7
PR-MP2	407.5	271.3	819.6	420.1	267.3	826.6
MP2.5	405.2	271.3	813.0	417.7	267.4	819.2
MP3	402.0	271.8	802.4	413.5	267.6	806.7
MP3.5	405.7	271.0	814.4	418.8	267.3	821.6
MP4(SDQ)	402.9	271.2	805.2	415.5	267.3	811.5

Table S4. Inter-chain IFIE sums from RBD for the closed structure (6VXX). Units in kcal/mol.

Method	6-31G*			cc-pVDZ		
	A (RBD)	B (RBD)	C (RBD)	A (RBD)	B (RBD)	C (RBD)
HF	-1375.5	-1429.8	-1368.8	-1388.0	-1439.4	-1388.4
MP2	-1645.1	-1707.8	-1634.9	-1696.4	-1757.2	-1694.3
PR-MP2	-1634.4	-1696.6	-1624.3	-1683.1	-1743.6	-1681.1
MP2.5	-1619.9	-1681.7	-1609.5	-1666.4	-1726.2	-1664.2
MP3	-1594.6	-1655.6	-1584.2	-1636.5	-1695.2	-1634.1
MP3.5	-1623.0	-1685.0	-1612.7	-1672.0	-1732.0	-1670.0
MP4(SDQ)	-1600.9	-1662.2	-1590.6	-1647.5	-1706.8	-1645.7

Table S5. Inter-chain IFIE sums from RBD for the open structure (6VYB). Units in kcal/mol.

Method	6-31G*			cc-pVDZ		
	A (RBD)	B (RBD)	C (RBD)	A (RBD)	B (RBD)	C (RBD)
HF	-944.6	-154.8	-983.5	-960.9	-160.8	-1008.7
MP2	-1136.6	-191.7	-1192.4	-1184.2	-207.4	-1252.9
PR-MP2	-1128.9	-190.2	-1184.1	-1174.5	-205.1	-1242.2
MP2.5	-1119.6	-188.6	-1173.7	-1163.1	-202.5	-1229.3
MP3	-1102.7	-185.5	-1155.0	-1141.9	-197.6	-1205.7
MP3.5	-1122.0	-189.0	-1176.2	-1167.4	-203.8	-1234.1
MP4(SDQ)	-1107.4	-186.3	-1160.1	-1150.6	-200.2	-1215.2

Table S6. Differential inter-chain IFIE sums from RBD between the closed structure (6VXX) and open structure (6VYB). Positive values indicate destabilization for the latter. Units in kcal/mol.

Method	6-31G*			cc-pVDZ		
	A (RBD)	B (RBD)	C (RBD)	A (RBD)	B (RBD)	C (RBD)
HF	430.9	1275.0	385.3	427.1	1278.7	379.7
MP2	508.6	1516.1	442.5	512.1	1549.8	441.4
PR-MP2	505.5	1506.4	440.3	508.5	1538.5	438.9
MP2.5	500.2	1493.1	435.8	503.4	1523.7	434.9
MP3	491.9	1470.1	429.2	494.6	1497.6	428.3
MP3.5	501.0	1496.0	436.5	504.6	1528.2	436.0
MP4(SDQ)	493.5	1476.0	430.5	497.0	1506.5	430.5

Table S7. Leading residues of chain-B RDB with large differences in IFIEs between the closed structure (6VXX) and open structure (6VYB). Values at MP3.5/cc-pVDZ level. Positive values indicate destabilization for the latter. Units in kcal/mol.

Residue	Closed Structure	Open Structure	ΔIFIE (Open - Closed)
Lys386	-286.2	-108.3	177.9
Lys378	-231.4	-58.3	173.2
Lys417	-223.2	-50.2	173.0
Lys458	-209.7	-44.2	165.5
Asp389	-69.0	94.5	163.4
Arg457	-199.2	-48.0	151.1
Lys424	-138.4	-56.9	81.6
Arg355	-130.3	-67.5	62.8
Arg408	-111.2	-52.1	59.1
Glu516	30.6	88.1	57.5

Table S8. IFIE sums between Spike RBD and ACE2 (6M0J). Units in kcal/mol.

	6-31G*	cc-pVDZ
HF	-714.6	-721.2
MP2	-832.6	-854.4
PR-MP2	-827.9	-848.8
MP2.5	-821.4	-841.2
MP3	-810.1	-828.0
MP3.5	-822.3	-842.5
MP4(SDQ)	-811.9	-830.6

Table S9. IFIE sums between Spike RBD and B38 Fab (7BZ5). Units in kcal/mol.

	6-31G*	cc-pVDZ
HF	-377.9	-363.6
MP2	-568.0	-576.7
PR-MP2	-560.0	-567.6
MP2.5	-549.7	-555.2
MP3	-531.4	-533.7
MP3.5	-551.6	-557.9
MP4(SDQ)	-535.2	-539.1

Table S10. Leading residues of Spike RDB in interactions with ACE2. Values at MP3.5/cc-pVDZ level. Units in kcal/mol.

Residue	IFIE
Lys417	-267.8
Arg403	-220.4
Arg408	-208.3
Lys444	-201.5
Arg509	-174.9
Lys378	-169.2
Arg457	-166.5
Arg454	-166.3
Lys458	-165.9
Arg346	-162.3

Table S11. PIEDA values between Spike RBD and ACE2 (6M0J). Units in kcal/mol, except for Distance in Å. Residue pairs with absolute IFIE values greater than 10 kcal/mol are listed. Asterisk specifies important interacting pair pointed out in the original paper on 6M0J (J. Lan et al., *Nature*, 2020, **581**, 215-220.).

Residue Pair (Spike RBD - ACE2)	Distance	IFIE	ES	EX	CT	DI	Ref
Lys417-Asp30	1.88	-119.48	-113.46	7.12	-8.21	-4.92	*
Lys417-His34	3.40	53.86	54.43	0.03	-0.17	-0.43	
Tyr449-Asp38	1.74	-35.24	-33.89	14.00	-9.66	-5.68	*
Glu484-Lys31	3.71	-66.26	-65.36	0.01	-0.47	-0.43	
Asn487-Gln24	1.82	-10.83	-14.91	13.69	-5.73	-3.88	*
Asn487-Tyr83	1.85	-11.12	-12.42	8.02	-4.40	-2.32	*
Phe497-Lys353	2.23	-20.42	-18.47	2.14	-2.25	-1.85	
Thr500-Asp355	2.97	-12.93	-8.53	0.25	-2.97	-1.68	
Asn501-Asp355	2.64	13.22	15.62	0.90	-1.56	-1.73	
Gly502-Gly354	1.77	-12.87	-17.86	13.94	-6.81	-2.13	*
Tyr505-Glu37	2.54	-13.19	-8.52	0.70	-3.03	-2.34	*

Table S12. Leading residues of Spike RDB in interactions with B38 Fab. Values at MP3.5/cc-pVDZ level. Units in kcal/mol.

Residue	IFIE
Asp420	-72.7
Lys417	-44.1
Asn487	-37.7
Gly476	-37.1
Arg403	-36.0
Asn501	-32.8
Tyr505	-32.5
Glu465	-24.2
Asp427	-23.4
Phe456	-23.3

Table S13. PIEDA values between Spike RBD and B38 Fab (7BZ5). Units in kcal/mol, except for Distance in Å. Residue pairs with absolute IFIE values greater than 10 kcal/mol are listed. Residues in parentheses indicate the real interacting residue via hydrogen bonding with the carbonyl oxygen atom, where this inconvenience was caused by the fragmentation at the C_α carbon atom of sp^3 type in usual FMO calculations; refer to our previous paper (R. Hatada et al., *J. Chem. Inform. Model.*, 2020, **60**, 3593-3602.) for details. "L" and "H" in parentheses for B38 Fab mean light and heavy chains, respectively. Two water molecules in the interface region have sizable interactions with charged residues.

Residue Pair (Spike RBD - B38 Fab)	Distance	IFIE	ES	EX	CT	DI
Arg403-Asn92 (Ser93) (L)	1.85	-32.45	-33.38	10.06	-5.93	-3.19
Asp405-Tyr94 (L)	2.91	-10.39	-6.80	2.44	-2.77	-3.25
Glu406-Water787	2.48	-16.31	-10.58	1.79	-4.60	-2.92
Thr415-Tyr58 (H)	1.77	-12.41	-13.28	12.37	-7.19	-4.31
Lys417-Tyr33 (H)	2.59	-11.18	-8.02	2.55	-2.02	-3.68
Lys417-Tyr52 (H)	2.11	-20.07	-15.64	5.46	-4.36	-5.52
Lys417-Glu98 (H)	4.82	-57.57	-57.45	0.00	-0.03	-0.08
Asp420-Ser56 (H)	1.50	-30.17	-44.18	35.31	-16.52	-4.78
Leu455 (Phe456)-Tyr33 (H)	1.64	-16.58	-22.10	18.02	-8.71	-3.79
Lys458-Ser30 (H)	2.41	-15.83	-12.31	2.03	-2.89	-2.65
Lys458-Ser31 (H)	1.73	-41.13	-42.95	16.78	-8.75	-6.21
Lys458-Asn32 (H)	3.20	11.80	11.85	0.24	0.41	-0.69
Lys458-Water402	2.50	-13.04	-10.01	3.80	-3.60	-3.23
Tyr473-Ser31 (Asn32) (H)	1.65	-12.59	-22.56	20.82	-9.01	-1.84
Ala475 (Gly476)-Ile28 (H)	1.99	-12.52	-11.29	7.23	-5.36	-3.10
Ala475 (Gly476)-Asn32 (H)	2.10	-14.51	-13.66	3.69	-2.95	-1.58
Asn487-Gly26 (Phe27) (H)	1.98	-11.15	-11.04	8.67	-5.39	-3.40
Asn487-Arg97 (H)	1.95	-28.42	-28.75	8.07	-4.57	-3.17
Tyr495 (Gly496)-Tyr32 (L)	2.37	-10.78	-8.48	1.22	-2.09	-1.43
Asn501-Ser30 (L)	1.88	-17.53	-16.40	9.34	-7.14	-3.33
Gly502-Ile29 (L)	1.80	-11.27	-16.64	13.36	-6.17	-1.82

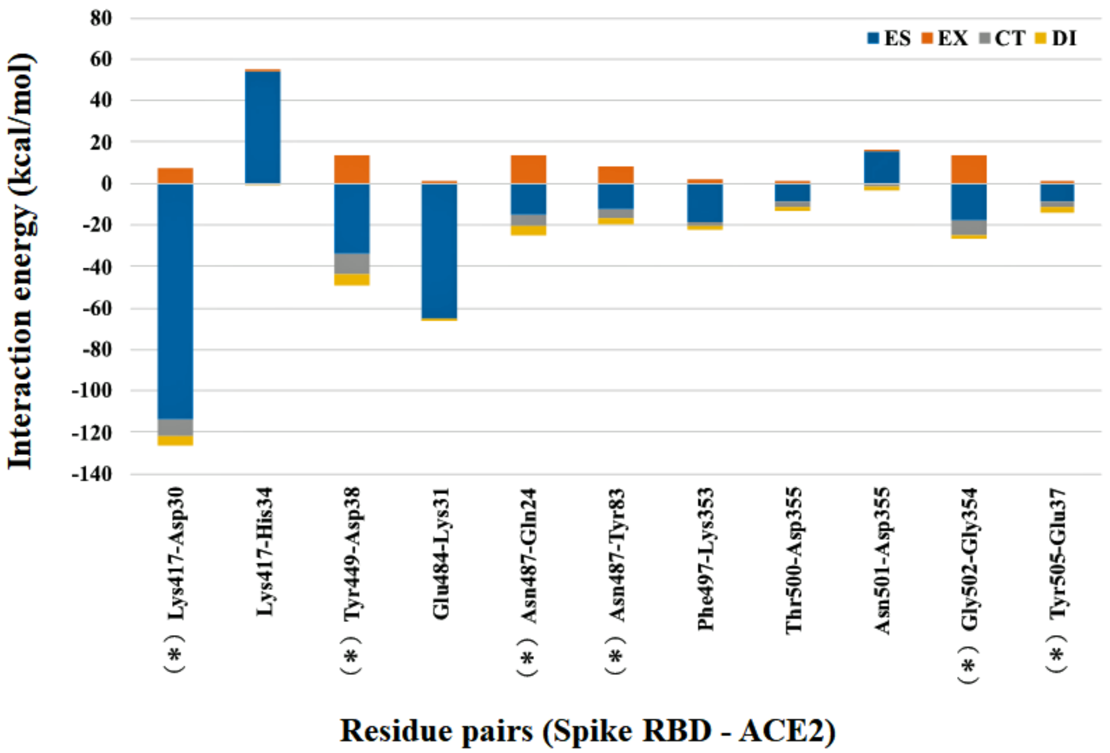

Figure S1. PIEDA results between Spike RBD and ACE2 (6M0J). Refer also to Table S11.

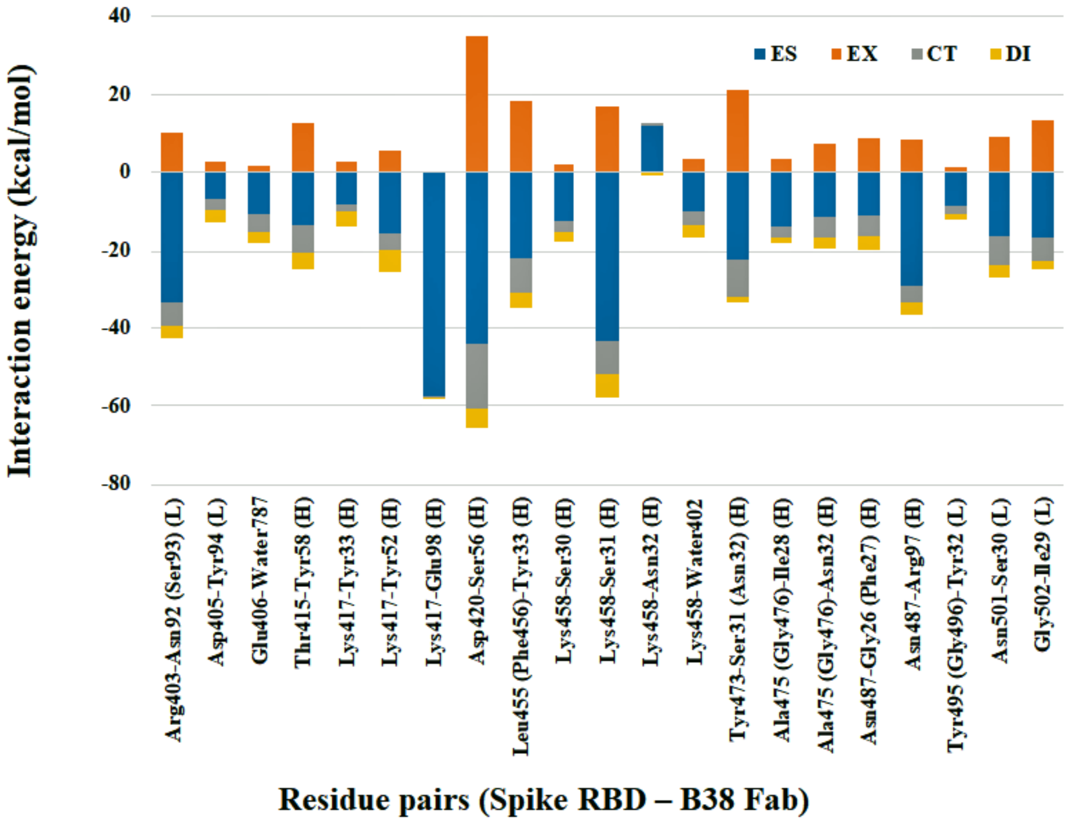

Figure S2. PIEDA results between Spike RBD and B38 Fab (7BZ5). Refer also to Table S13.
